# Ultrasonographic Appearance of a Posterior Lenticonus in a Cat

**DOI:** 10.1111/vop.70024

**Published:** 2025-05-16

**Authors:** Antonella Rampazzo, Marc Orts‐Porcar, Francesca Del Chicca

**Affiliations:** ^1^ Equine Department, Section Ophthalmology Vetsuisse Faculty, University of Zurich Zurich Switzerland; ^2^ Clinic for Diagnostic Imaging Vetsuisse Faculty, University of Zurich Zurich Switzerland

**Keywords:** cat, cataract, lenticonus, posterior lens capsule, posterior lens capsule rupture, ultrasonography

## Abstract

The objective of this study is to present high‐quality and up‐to‐date ocular ultrasonographic images and videos of a posterior lenticonus with concomitant mature cataract in a cat. Additionally, the clinical findings, surgical treatment, and outcome are reported for completeness and to confirm the diagnosis. An 8‐year‐old castrated male domestic shorthair cat presented due to unilateral cataract. Ophthalmological examination revealed a mature cataract without clinically detectable uveitis. Hematological and biochemical analyses were unremarkable, and tests for common infectious diseases associated with feline uveitis were negative. Electroretinography and ocular ultrasound were performed as part of the presurgical planning for phacoemulsification. Retinal function was normal. Ultrasonographic examination revealed a severely distorted shape of the posterior lens capsule (PLC) with hyperechoic material protruding into the vitreous body. Neither a PLC fibrovascular plaque nor a persistent hyaloid artery was identified. These findings were interpreted as a possible rupture of the posterior lens capsule with lens material extruding into the vitreous body. Posterior lenticonus was considered a less likely differential diagnosis. The owner elected to proceed with phacoemulsification surgery. Intraoperatively, the posterior lens capsule was found to be intact. An axial posterior lenticonus with cataractous lens material bulging together with the capsule into vitreous was identified. An intraocular lens was successfully implanted. Given the better surgical prognosis compared to capsule ruptures, posterior lenticonus, although rare, should be considered a differential diagnosis, particularly when a cone‐shaped protrusion lacking Doppler signal is identified at the posterior lens capsule extending into the vitreous body.

## 1 Introduction

Posterior lenticonus is a congenital malformation of the lens characterized by a localized, round to oval, well‐circumscribed protrusion of the posterior lens capsule and cortex [1]. Instead of having a smoothly convex surface, the posterior cortical and capsular regions have a circumscribed cone‐like (lenticonus) or globular (lentiglobus) protrusion of variable size [2]. The widely accepted hypothesis states that the posterior lenticonus develops by herniation of cortical lens fibers and the posterior lens capsule into the vitreous at an area of posterior lens capsule weakness during fetal development [3]. The presumed cause for the development of cataracts in the posterior lenticonus is mechanical, due to posterior bowing of the capsule and progressive degeneration of lens fibers. The opacity usually starts at the posterior pole and may progress rapidly to involve the whole lens [4]. Posterior lenticonus has been described in humans, rabbits, mice, cattle, calves, dogs, and cats [2, 5, 6]. Posterior lenticonus alone or in combination with cataract and/or other multiple congenital anomalies has been reported in several dog breeds, including Miniature Schnauzer and Bull Mastiff, Cavalier King Charles Spaniel, Akita Inu, and Shih Tzu [2, 7, 8, 9]. Lenticonus may be unilateral or bilateral and has been reported both with an intact lens capsule and, in other cases, with histologic evidence of lens capsule rupture [7]. In breeds, such as Doberman Pinscher, Golden Retriever, Bouvier des Flandres, Bloodhound, Old English Sheepdog, Labrador Retriever, Staffordshire Bull Terrier, and American Staffordshire Terrier, lenticonus and/or lentiglobus have also been associated with retrolental fibrovascular plaques, microphthalmia, coloboma, retinal dysplasia, optic nerve hypoplasia, and intraocular hemorrhage [10, 11, 12, 13, 14, 15, 16, 17]. Lenticonus is a feature of persistent hyperplastic tunica vasculosa lentis/persistent hyperplastic primary vitreous (PHTVL/PHPV) anomaly grades 4–6 [10, 18].

With the exception of cataracts, congenital malformations of the lens are rare in cats [19]. In 1983, Peiffer et al. [20] reported a case of keratolenticular dysgenesis in a kitten. In 2015, Bauer et al. [5] described a case of a cat with bilateral anterior segment dysgenesis, incipient cataract, and anterior and posterior lenticonus. In 2001, Allgoewer et al. reported two feline patients with severe lenticular and retrolental pathology resembling grade 5 of Stades' grading scheme for PHTVL/PHPV in the Doberman pinscher [10, 21]. In 1998, Barnett et al. [22] documented a Ragdoll kitten affected with unilateral persistent hyaloid artery. Considering the literature, feline posterior lenticonus appears to be a rare abnormality. In pediatric ophthalmology, lenticonus is an uncommon congenital abnormality, often associated with cataract, that poses a surgical challenge as the presentation varies from protruding lens material accompanied by a thin, bulging posterior capsule to large, preexisting posterior capsular dehiscence [23]. Veterinary ophthalmologists are more familiar with the similar condition in dogs. In this species, lenticonus might be part of PHTVL/PHPV (grades 4–6), or present alone with or without posterior lens capsule plaques or with persistent hyaloid artery.

In the 1970s and 1980s, several high‐quality reports were published on the clinical and histopathological findings of PHTVL/PHPV and posterior lenticonus in dogs [2, 7, 8, 10]. In the 1990s and early 2000s, additional studies included ultrasonographic ocular examinations alongside clinical findings. Using 7.5–10 MHz probes, various authors documented ultrasonographic images of PHTVL/PHPV, persistent hyaloid artery, and posterior lenticonus in dogs [9, 14, 24]. Regarding posterior lenticonus in cats, only two images of one case have been reported as part of a two‐case series published by Allgoewer et al. [21]. The ultrasound images in that study were obtained with a 10 MHz linear probe, representing the highest quality achievable with the technology available at that time.

Since these pathologies are well documented, relatively few new publications are available, leading to a lack of ultrasonographic images provided using the state‐of‐the‐art equipment. Modern ultrasonographic machines with high frequency probes (14–18 MHz) provide much more detailed images with increased spatial resolution compared to older technology. An exception is the ultrasonographic and intraoperative images of lens posterior capsular ectasia in a dog, published by Pizzirani [25].

The aim of this case report is to present high‐quality, up‐to‐date ocular ultrasonographic images and videos of posterior lenticonus in cats, a rare condition with limited detailed imaging reports. The images were obtained using a wideband linear array ultrasound probe with working frequencies ranging between 14 and 18 MHz, allowing for increased spatial resolution and detail. Accurate interpretation of ultrasonographic findings can be challenging, particularly in the presence of posterior lens capsule deformity. In such cases, ophthalmologists and radiologists may be more inclined to suspect posterior lens capsule rupture rather than a rare condition like lenticonus. However, as the surgical prognosis for lenticonus is more favorable than for posterior capsule rupture, precise imaging interpretation and recognition of this condition are crucial for optimal patient outcomes.

## 2 Case Report

An 8–year‐old castrated male domestic shorthair cat was referred to the Ophthalmology Section of the Vetsuisse Faculty at the University of Zurich due to unilateral cataract. The owner noted the lens opacity for the first time 3–4 months prior to referral. The cat lived exclusively indoors with no other pets. The owner reported no known trauma, no ocular pain, and stated that the eye appeared open and comfortable at home. The cat was otherwise healthy. The referring veterinarian performed a complete hematochemistry, including testing for infectious diseases and hyperthyroidism before referral. Bloodwork was unremarkable, and T4 was within normal limits. Toxoplasma IgG and IgM antibodies (IFT), FeLV‐FIV (rapid FeLV Ag test, FIV ELISA), and Encephalitozoon cuniculi antibodies (IFAT) were negative. A complete ophthalmic examination was performed. Instruments used included a slit lamp biomicroscope (SL‐19, Kowa, Japan), an indirect ophthalmoscope (Heine Optotechnik, Germany), and a rebound tonometer (Tonovet Type TV01). Examination of the right eye (OD) revealed a severely reduced menace response, positive dazzle, and direct PLR reflexes. Eyelids, conjunctiva, and cornea were unremarkable. The anterior chamber was clear (no detectable flare or cells with 10 and 16× slit lamp magnification) and the iris presented a focal pupillary margin eversion (ectropion uvea). After mydriasis obtained with 0.5% tropicamide (Tropicamide Opth 0.5%, Théa PHARMA), the lens was examined. Diffuse mature cataract was diagnosed. The vitreous body and fundus were not assessable. Examination of the left eye (OS) revealed a positive menace response, positive dazzle, and direct PLR reflexes. Eyelids, conjunctiva, cornea, anterior chamber, and iris were within normal limits. The lens was examined after pupil dilation, and prominent anterior and posterior suture lines could be seen. The vitreous body and fundus were unremarkable. Schirmer Tear Test (Schirmer Tear Test Standardized Sterile Strips, MSD Animal Health) was 15 mm/min in both eyes (OU). Fluorescein Testing (Fluo strips) was negative OU. Intraocular pressure was 11 mmHg OD and 15 mmHg OS. A tentative diagnosis of complete mature cataract without evident signs of uveitis was made. Bilateral ocular ultrasound examination (B scan, 14.0–18.0 MHz Probe L8‐18i, GE Vet, LOGIQ E10, General Electric HealthCare Company, USA) and electroretinography (ERG) (RETIport 3S electroretinography, An‐Vision, Germany) were performed under sedation with butorphanol 0.2 mg/kg (Alvegesic 1% Virbac) and 5 μg/kg medetomidine (Medetor Virbac) IM and after administration of topical anesthesia of the ocular surface achieved with oxybuprocaine hydrochloride (Oxybuprocaine 0.4%, Thea Pharma) and mydriatic eye drops.

**FIGURE 1:**
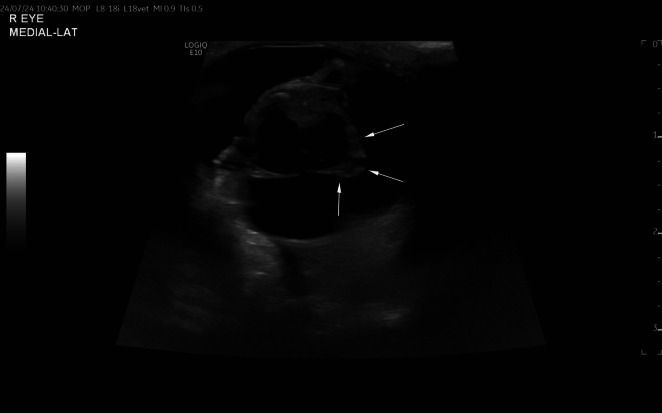
Transcorneal axial image from the ultrasonographic study of the right eye. The arrows delineate the margins of the posterior lenticonus. Note the abnormal conical shape of the posterior aspect of the lens with protrusion into the vitreous body. The increased echogenicity of the lens can just be seen partially in this image (asterisk) but better appreciated in the Video 1.

Transcorneal B‐mode ultrasonographic examination of both eyes was performed using an electronic wideband linear array (L8‐18i) probe, with frequencies ranging between 14 and 18 MHz. The ultrasonographic examination of the right eye revealed significant lens abnormalities in shape and echogenicity (Figure 1). The right lens displayed markedly increased echogenicity. The anterior capsule was normal, but the posterior lens capsule was abnormally shaped. It was irregularly thickened with a cone‐shaped protrusion of the axial portion into the vitreous body. This conical protrusion had broad‐based contact with the posterior lens capsule, a mildly irregular surface, and heterogeneous hyperechogenicity. It extended approximately 5 mm into the vitreous body. No Doppler signal was detected, suggesting the absence of vascular involvement. No structures connecting the lens to the optic nerve were visible. The axial lens thickness, including the conical protrusion, measured 9.5 mm, while the lens equatorial diameter measured 12.3 mm. A video of the ultrasonographic study of the right eye is available (Video 1). The ultrasonographic examination of the left eye was within normal limits. Considering the ultrasonographic findings, the presumptive diagnosis was a rupture of the posterior lens capsule. Differential diagnoses included posterior lenticonus and PHTVL/PHPV. A neoplastic process or a patent persistent hyaloid artery was considered less likely given the absence of color Doppler signal and other concomitant findings.

**VIDEO 1:** The video of the ultrasonographic study of the right eye illustrates the posterior lenticonus. Note the abnormal shape of the lens, with posterior conic‐shape protrusion into the vitreous body. Also, note the increased echogenicity in the lens, consistent with a mature cataract. Video content can be viewed at https://onlinelibrary.wiley.com/doi/10.1111/vop.70024

The retinal function was assessed with a dark‐adapted, mixed rod‐cone flash ERG [26]. The a‐wave implicit time was 14 ms OD and 11 ms OS; the b‐wave implicit time was 35 ms OD and 34 ms OS. The b‐wave amplitude was 270 μV OD and 340 μV OS.

Findings and potential risk factors for surgical cataract removal were discussed with the owner. The suspicion of posterior lens capsule rupture, with lens material extruding into the vitreous, was a major concern. Given the poor to absent vision in the affected eye and the potential to restore it, the owner, with support from the referring veterinarian, opted for phacoemulsification.

Cataract extraction OD was performed with routine phacoemulsification under general anesthesia, following the administration of IM premedication, using methadon 0.2 mg/kg (Methadon Streuli) and dexmethomidine 5 μg/kg (Dexdomitor). The cat had an IV catheter placed and was induced using a combination of IV ketamine 1 mg/kg (Ketnarkon 100) and alfaxalone 0.5 mg/kg (Alfaxan). The cat was maintained under sevoflurane inhalant anesthesia, and muscular relaxation was achieved using 0.4 mg/kg rocuronium bromide IV (Esmeron) and additional 0.2 mg/kg doses as needed throughout anesthesia. The eye was prepared for ophthalmic surgery using a 2% povidone iodine flush (NaCl 0.9%, Fresenius Kabi, Betadine) and two to three drops of topical anesthesia (Oxybuprocaine 0.4%, Thea Pharma) applied afterward. After a clear corneal incision of 2.8 mm (MSL28 slit‐angled 2.8 mm Ophthalmic Knife, Mani Inc.) was made, the anterior chamber (AC) was injected with approximately 0.1 mL of adrenalin (Adrenalin Sintetica 1 mg/mL Ampullen, Sintetica, CH) to achieve mydriasis, the anterior lens capsule was stained with Trypan blue (an‐blue, An‐Vision, Germany) and then the AC was filled with high‐viscosity 1.8% sodium hyaluronate (an‐bfh 1.8%, An‐vision, Germany). An anterior capsulorhexis of approximately 5 mm was performed with high‐frequency capsulotomy (Capsultomy tip, Oertli Switzerland) and Utrata forceps. Hydro‐dissection was avoided to prevent extension of the suspected posterior capsular tear. Routine one‐handed phacoemulsification was carried out. Bimanual technique and bimanual I/A and vitrectomy probe were ready to use in case conversion of the one‐handed technique would have been necessary. The nucleus and the adjacent cortex were deemed dense cataracts. Part of the posterior cortex was bulging posteriorly into an axial area of distended and extremely thin posterior lens capsule (PLC). The capsule was intact and with very careful one‐handed irrigation/aspiration (I/A) the cortex was removed uneventfully. The part of the posterior cortex bulging into the vitreous was soft and therefore it was possible to gently aspirate it with I/A. The axial distended part of the PLC was fluttering during I/A. In human medicine, this phenomenon is described as jellyfish or fishtail sign, indicating this unusual focal blowing curtain effect of the PLC. Since the PLC was clear, posterior capsulotomy was not performed. After enlarging the corneal incision to 4.1 mm with a clear corneal knife (Implant Slit Knife 4.1 mm angled round tip, Ophthalmic Knife, Mani Inc.), a feline acrylic intraocular lens (IOL) of +53.5 D (MC1‐13 An‐lens, An‐vision, Germany) was implanted in the lens capsular bag. After viscoelastic removal, the corneal incision was apposed using 9–0 polyglactin 910 (Ethicon Coated Vicryl) in a simple interrupted pattern. Before completing the corneal suture, intracameral carbachol 0.1 mL (Miostat 0.1 mg/mL, Alcon, CH) and cefuroxime 1 mg (0.1 mL) (ceFUROxime Labatec 750 mg reconstituted using NaCl 0.9%, Fresenius Kabi) were administered [27, 28]. Following surgery, pain control was achieved with methadon 0.2 mg/kg IV (Methadon 10 mg/mL, Sintetica SA). Postoperative therapy of the right eye included ofloxacin QID (Floxal opht 0.3%, Bausch & Lomb Swiss AG), ketorolac BID (Acular opht, AbbVie AG), prednisolone TID (Pred Forte opht 1%, AbbVie), and an ocular lubricant QID (Ocry‐gel, Virbac). Systemic cefalexin (Cefaseptin, Vetoquinol AG, Bern) 20 mg/kg BID PO for 1 week and prednisolone (Hedylon Graeub) at 1 mg/kg PO q24 h for 1 week, then 0.5 mg/kg for 1 week, then 0.25 mg/kg for 1 week were administered. At the 1‐day postsurgery recheck, the eye appeared comfortable, menace response was positive, and dazzle and PLR reflexes were positive. Corneal sutures were not leaking. The AC presented trace flare, the IOL was centered, and the vitreous body and fundus were unremarkable. Since IOP was 17 mmHg in the operated eye and OS could not be measured due to low compliance of the cat, it was considered safe to start brinzolamide BID (Azopt susp opht, Novartis Pharma, Schweiz AG) topically to prevent possible IOP elevation. At the 1‐week recheck, the eye was still comfortable, visual, and healing uneventfully. The only complication was the persistence of trace flare. At this recheck, the IOP was 9 mmHg (OS could not be measured due to low compliance of the cat) and brinzolamide eye drops were discontinued. The other medications were continued as indicated above, except for ketorolac which was stopped. At the 2 weeks recheck, the eye was visual still showing mild uveitis, with detectable flare in the AC ranging from traces to 1+, slight miosis and an IOP of 7 mmHg (IOP OS 20 mmHg). Ofloxacin eye drops were discontinued and the topical and systemic prednisolone were continued to the next recheck.

The cat was scheduled for rechecks at 1, 2, and 4 months postoperatively. Ocular lubricants were continued until the 4‐month recheck, while topical prednisolone was discontinued at the 2‐month recheck and replaced with ketorolac q24h until the 4‐month recheck. At the final follow‐up, 5 months postoperatively, the eye had been untreated for 1 month and remained visual, free from inflammation, and without other complications.

## 3 Discussion

According to the literature reviewed during the present study, this is the first report of posterior lenticonus in a cat documented with the image quality and achievable detail using current ultrasonographic equipment. As this is a rare condition, it is possible to come across only a few cases throughout a professional career. Given that this pathology in dogs is often associated with PHTVL/PHPV, with or without a persistent hyaloid artery, the authors believe that familiarity with its ultrasonographic appearance in cats is valuable. In the authors' opinion, such familiarity would facilitate accurate diagnosis and treatment. The most likely differential diagnosis was a rupture of the posterior lens capsule, although the complete absence of clinically detectable uveitis did not fully support this hypothesis. Lens capsular tears in veterinary medicine are often secondary to trauma, particularly sharp trauma (e.g., cat claw), or to swelling and an increase in size of the lens fibers, which is most commonly seen in diabetic cataracts but can also occur in other types of cataracts. Traumatic lens capsule ruptures are mostly seen at the anterior lens surface, as claws or foreign bodies typically penetrate through the cornea. In such cases, the laceration can often be directly observed via biomicroscopy during an ophthalmological examination. If the sharp injury occurs through the limbus, the lens capsule may rupture near the lens equator. Primary cataracts, when markedly swollen, or diabetic cataracts, which are often intumescent, can also cause lens rupture. These ruptures typically occur at the equator or the posterior capsule. In the case of PLC rupture, ultrasonographic differentiation from lenticonus can be challenging, as both conditions present hyperechoic material protruding in the vitreous body with associated irregular margins of the lens capsule. The video presented in this case report highlights the ultrasonographic features of posterior lenticonus, as confirmed during surgery. Recognizing posterior lenticonus with the integrity of the posterior lens capsule is essential because of the better prognosis compared to cases involving rupture due to trauma or cataracts. On the other hand, when deformation of the posterior capsule is present, ultrasonographic assessment does not always allow differentiation between an irregular or thickened, yet intact capsule with bulging lens material (as seen mostly in lenticonus) and an actual loss of capsule integrity with extrusion of lens contents into the vitreous body (as occurs in rupture).

In the present case, the absence of the Doppler signal in the protruding material and the lack of structures connecting the lens to the optic nerve further supported a nonvascular process, reducing the likelihood of a persistent hyaloid artery, a persistent hyperplastic tunica vasculosa lentis, or a neoplastic process.

In 2001, Dr. Allgoewer described PHTVL/PHPV in two young cats, where, due to the low chances for regaining vision, the owner declined surgery [21]. In the case we presented, the PLC visualized intraoperatively was intact and clear, without an axial plaque like that seen in dogs affected by PHTVL/PHPV grade 4 or more [18]. In cases of lens capsule rupture, the development of secondary phacoclastic uveitis is typically expected, depending on the size of the rupture and the species affected. This was not observed in our patient and led us to the other differential diagnoses previously discussed for the ultrasonographic findings. Conversely, lenticonus is a congenital malformation that is usually identified earlier in life and not typically in an 8‐year‐old adult cat, making it a lower priority on the clinical differential diagnosis list. In humans, visual acuity reduction due to lenticonus is a commonly reported complaint in children. Some cases may develop cataracts associated with lenticonus, which can be rapidly progressive (weeks to months) [4, 29, 30]. In veterinary patients, early signs of lenticonus may go unnoticed, as owners are unlikely to detect subtle visual deficits. Consequently, affected animals might be more likely to present later in life if concurrent cataracts develop.

In human medicine, posterior polar and posterior lentiglobus cataracts represent a large subset of nontraumatic cataracts in pediatric cataract specialty practice. Visual outcomes are generally good, and complications are rare [31]. On the other hand, it is described that posterior lenticonus can result in rupture of the PLC, and even if this is not the case, the weakness of the posterior capsule will make the surgery challenging [32]. The bulge in the posterior capsule can be appreciated intraoperatively as a flutter. This movement becomes very noticeable during aspiration of the cortical material and increases with chamber instability [33]. This effect is called by some authors “jellyfish” or “fish‐tail” sign [34, 35]. Dr. Wilson reports in his case series that the area of capsule bulging varied in size from 2 mm to 7 mm and varied in location from central to paracentral. Therefore, even eyes with an intact posterior capsule are at higher risk of intraoperative rupture, and appropriate surgical maneuvering must be performed to avoid intraoperative rupture. Some eyes might present with a preexisting rupture of the posterior capsule; therefore, hydrodissection is contraindicated in cases of cataract with known or suspected posterior lentiglobus. Hydrodissection leads to a sudden buildup of hydraulic pressure, which can cause uncontrolled tearing of the posterior capsule at the lentiglobus defect, sending lens material into the vitreous and threatening the capsular bag stability [32]. In human ophthalmology, it is advised that the presence of a possible ruptured posterior capsule shall be examined by vigilant inspection at the beginning of phacoemulsification. In the absence of posterior capsule dehiscence, the peripheral cortex shall be aspirated first, followed by central lens matter aspiration like “outside‐in.” Contrarily, if a tear of the PLC is noted, the surgeon should change the direction of lens matter aspiration by aspirating the central lenticular matter first, followed by peripheral cortex like “inside‐out.” [23] Considering, as reported in human pediatric ophthalmology, that lenticonus can in some cases be accompanied by posterior lens capsule rupture, and that ultrasonographic differentiation between an intact and a ruptured posterior lens capsule is challenging in both traumatic and lenticonus cases, a surgical careful approach is always recommended.

## 4 Conclusion

The authors display high‐quality images of the ultrasonographic appearance of a posterior lenticonus with an intact posterior lens capsule, accompanied by a mature cataract, in a cat. Although rare in this species, posterior lenticonus should be recognized and considered a possible differential diagnosis when a cone‐shaped protrusion of the posterior lens capsule into the vitreous body is observed. In this case, the owner and the referring veterinarian were highly motivated to pursue surgery despite the unfavorable prognosis due to the presumptive diagnosis of a posterior lens capsule rupture. The surgery proceeded uneventfully, and vision was restored.

## Author Contributions

**Antonella Rampazzo:** conceptualization, investigation, writing – original draft, methodology, writing – review and editing, project administration, supervision. **Marc Orts‐Porcar:** writing – original draft, visualization, writing – review and editing, investigation, methodology. **Francesca Del Chicca:** investigation, writing – original draft, methodology, visualization, writing – review and editing, supervision.

## Ethics Statement

Animal owners or owners' representatives provided written informed consent for the procedure and therapy undertaken and publication of data and images (pdf file “Owner informed consent”).

## Conflicts of Interest

The authors declare no conflicts of interest.

## Data Availability

The data that support the findings of this study are available from the corresponding author upon reasonable request.

## References

[1] M. L. Gibbs , M. Jacobs , A. O. Wilkie , and D. Taylor , “Posterior Lenticonus: Clinical Patterns and Genetics,” Journal of Pediatric Ophthalmology and Strabismus 30, no. 3 (1993): 171–175, 10.3928/0191-3913-19930501-10. 8350227

[2] G. Aguirre and S. I. Bistner , “Posterior Lenticonus in the Dog,” Cornell Veterinarian 63, no. 3 (1973): 455–461. 4782563

[3] M. Khalil and N. Saheb , “Posterior Lenticonus,” Ophthalmology 91, no. 11 (1984): 1429–1443, 10.1016/s0161-6420(84)34132-x. 6514312

[4] L. Amaya , D. Taylor , I. Russell‐Eggitt , K. K. Nischal , and D. Lengyel , “The Morphology and Natural History of Childhood Cataracts,” Survey of Ophthalmology 48, no. 2 (2003): 125–144, 10.1016/s0039-6257(02)00462-9. 12686301

[5] B. S. Bauer , L. S. Sandmeyer , and B. H. Grahn , “Diagnostic Ophthalmology,” Canadian Veterinary Journal 56, no. 5 (2015): 519–520. PMC4399743 25969589

[6] S. Osinchuk , L. Petrie , M. Leis , et al., “Congenital Nuclear Cataracts in a Holstein Dairy Herd,” Canadian Veterinary Journal 58, no. 5 (2017): 488–492. PMC5394606 28507388

[7] K. Narfström and R. Dubielzig , “Posterior Lenticonus, Cataracts and Microphthalmia; Congenital Ocular Defects in the Cavalier King Charles Spaniel,” Journal of Small Animal Practice 25 (1984): 669–677, 10.1111/j.1748-5827.1984.tb03380.x.

[8] L. J. Laratta , R. C. Riis , T. J. Kern , and S. A. Koch , “Multiple Congenital Ocular Defects in the Akita Dog,” Cornell Veterinarian 75, no. 3 (1985): 381–392. 3926378

[9] J. I. Ori , T. Yoshikai , S. Yoshimur , H. Ujino , and K. Takase , “Posterior Lenticonus With Congenital Cataract in a Shih Tzu Dog,” Journal of Veterinary Medical Science 62, no. 11 (2000): 1201–1203, 10.1292/jvms.62.1201. 11129866

[10] F. C. Stades , “Persistent Hyperplastic Tunica Vasculosa Lentis and Persistent Hyperplastic Primary Vitreous (PHTVL/PHPV) in 90 Closely Related Doberman Pinschers: Clinical Aspects,” Journal of the American Animal Hospital Association 16 (1980): 739–751.

[11] J. S. Van der Linde‐Sipman , F. C. Stades , and D. de Wolff‐Rouendaal , “Persistent Hyperplastic Tunica Vasculosa Lentis and Persistent Hyperplastic Primary Vitreous in the Doberman Pinscher: Pathological Aspects,” Journal of the American Animal Hospital Association (USA) (1983).

[12] J. D. Lavac and G. A. Severin , “Posterior Lenticonus and Lenticonus Internum in a Dog,” Journal of the American Animal Hospital Association 13 (1977): 685–867.

[13] I. B. J. Van Rensburg , S. W. Petrick , and M. E. E. Van der Lugt Smit , “Multiple Inherited Eye Anomalies Including Persistent Hyperplastic Tunica Vasculosa Lentis in Bouvier de Flandres,” Progress in Veterinary and Comparative Ophthalmology 2 (1992): 133–139.

[14] A. J. Gemensky‐Metzler and D. A. Wilkie , “Surgical Management and Histologic and Immunohistochemical Features of a Cataract and Retrolental Plaque Secondary to Persistent Hyperplastic Tunica Vasculosa Lentis/Persistent Hyperplastic Primary Vitreous (PHTVL/PHPV) in a Bloodhound Puppy,” Veterinary Ophthalmology 7, no. 5 (2004): 369–375, 10.1111/j.1463-5224.2004.04032.x. 15310298

[15] K. P. Barrie , R. L. Peiffer , K. N. Gelatt , and L. W. Williams , “Posterior Lenticonus, Microphthalmia, Congenital Cataracts, and Retinal Folds in an Old English Sheepdog,” (1979), 715–717.

[16] R. Curtis , K. C. Barnett , and A. Leon , “Persistent Hyperplastic Vitreous in the Staffordshire Bull Terrier,” Veterinary Record 115 (1984): 385. 6506414 10.1136/vr.115.15.385

[17] A. Leon , B. Curtis , and K. C. Barnett , “Hereditary Persistent Hyperplastic Primary Vitreous in the Staffordshire Terrier,” Journal of the American Animal Hospital Association 22 (1986): 765–774.

[18] M. H. Boeve and F. C. Stades , “Diseases and Surgery of the Canine Vitreous,” in Veterinary Ophthalmology Textbook, vol. II, 6th ed., ed. K. N. Gelatt (Wiley‐Blackwell, 2021), 1467.

[19] M. B. Glaze , D. J. Maggs , and C. E. Plummer , “Feline Ophthalmology,” in Veterinary Ophthalmology Textbook, vol. II, 6th ed., ed. K. N. Gelatt (Wiley‐Blackwell, 2021), 1774.

[20] R. L. Peiffer, Jr. and P. V. Belkin , “Keratolenticular Dysgenesis in a Kitten,” Journal of the American Veterinary Medical Association 182, no. 11 (1983): 1242–1243, 10.2460/javma.1983.182.11.1242. 6863142

[21] I. Allgoewer and B. Pfefferkorn , “Persistent Hyperplastic Tunica Vasculosa Lentis and Persistent Hyperplastic Primary Vitreous (PHTVL/PHPV) in Two Cats,” Veterinary Ophthalmology 4, no. 2 (2001): 161–164, 10.1046/j.1463-5224.2001.00177.x. 11423000

[22] K. C. Barnett and S. M. Crispin , Feline Ophthalmology: An Atlas and Text (Saunders, 1998).

[23] G. Chattannavar and R. Kekunnaya , “Inside‐Out and Outside‐In: Tips and Tricks in Posterior Lenticonus,” Indian Journal of Ophthalmology 70, no. 9 (2022): 3431, 10.4103/ijo.IJO_903_22. PMC9675546 36018145

[24] S. A. Boroffka , A. M. Verbruggen , M. H. Boevé , and F. C. Stades , “Ultrasonographic Diagnosis of Persistent Hyperplastic Tunica Vasculosa Lentis/Persistent Hyperplastic Primary Vitreous in Two Dogs,” Veterinary Radiology & Ultrasound 39, no. 5 (1998): 440–444, 10.1111/j.1740-8261.1998.tb01632.x. 9771597

[25] D. Penninck and M.‐A. d'Anjou , Atlas of Small Animal Ultrasonography, 2nd ed. (Wiley Blackwell, 2015) Chap.2 Pizzirani, Penninck and Spaulding.

[26] 26. B. Ekesten , A. M. Komáromy , R. Ofri , S. M. Petersen‐Jones , and K. Narfström , “Guidelines for Clinical Electroretinography in the Dog: 2012 Update,” Documenta Ophthalmologica 127 (2013): 79–87, 10.1007/s10633-013-9388-8. 23728902

[27] G. M. Keating , “Intracameral Cefuroxime: Prophylaxis of Postoperative Endophthalmitis After Cataract Surgery,” Drugs 73, no. 2 (2013): 179–186, 10.1007/s40265-013-0011-9. 23338537

[28] P. Nucci , A. Lembo , I. Schiavetti , F. Rissotto , and F. Pichi , “Exploring the Safety and Feasibility of Intracameral Aprokam (Cefuroxime Sodium) in Pediatric Cataract Surgery,” International Ophthalmology 45, no. 1 (2025): 25, 10.1007/s10792-024-03404-2. 39831915

[29] B. G. Mohney and M. M. Parks , “Acquired Posterior Lentiglobus,” American Journal of Ophthalmology 120 (1995): 123–124. 7611322 10.1016/s0002-9394(14)73774-2

[30] R. Kekunnaya , A. V. Deshmukh , and S. Kulkarni , “Newer Insights Into the Clinical Profile of Posterior Lenticonus in Children and Its Surgical, Visual, Refractive Outcomes,” Eye 36 (2022): 985–993, 10.1038/s41433-021-01564-4. 33958736 PMC9046400

[31] S. K. Mistr , R. H. Trivedi , and M. E. Wilson , “Preoperative Considerations and Outcomes of Primary Intraocular Lens Implantation in Children With Posterior Polar and Posterior Lentiglobus Cataract,” Journal of AAPOS 12, no. 1 (2008): 58–61, 10.1016/j.jaapos.2007.08.003. 18029213

[32] M. E. Wilson and R. H. Trivedi , “Intraocular Lens Implantation in Pediatric Eyes With Posterior Lentiglobus,” Transactions of the American Ophthalmological Society 104 (2006): 176–182. 17471338 PMC1809921

[33] M. R. Praveen , A. R. Vasavada , A. Koul , et al., “Intraoperative Posterior Capsule Flutter in Posterior Lentiglobus,” Journal of AAPOS 14, no. 4 (2010): 367–368, 10.1016/j.jaapos.2010.02.008. 20598928

[34] S. Ganesh , S. Brar , and K. Chopra , “Jellyfish Sign for Intraoperative Identification of Posterior Lenticonus,” International Ophthalmology 37, no. 5 (2017): 1239–1241, 10.1007/s10792-016-0386-1. 27798716

[35] Y. Ding , J. Zhang , and Y. Huang , “Influence of Posterior Capsule Abnormalities in Pediatric Cataract Surgery,” Journal of Cataract and Refractive Surgery 50, no. 2 (2024): 146–152, 10.1097/j.jcrs.0000000000001324. 37816250 PMC10805350

